# Severe alveolar bone resorption in Felty syndrome: a case report

**DOI:** 10.1186/s13256-022-03703-1

**Published:** 2022-12-16

**Authors:** Satoru Morikawa, Yoko Miyashita, Mana Nasu, Shunichi Shibazaki, Satoshi Usuda, Kazuyuki Tsunoda, Taneaki Nakagawa

**Affiliations:** Department of Dentistry and Oral Surgery, Keio University School of Medicine, 35 Shinanomachi, Shinjuku-Ku, Tokyo, 160-8582 Japan

**Keywords:** Felty syndrome, Periodontitis, Pancytopenia, Neutropenia, Oral hygiene, Alveolar bone resorption, Infection, Case report, Anemia, Periodontal treatment

## Abstract

**Background:**

Felty syndrome is defined by three conditions: neutropenia, rheumatoid arthritis, and splenomegaly. Neutropenia associated with pancytopenia may further affect the dental condition of a patient. Periodontal treatment and surgery in patients with Felty syndrome necessitates cooperation with a hematologist. Here we present a case of a patient with Felty syndrome who was initially referred to the oral surgery hospital attached to the School of Dentistry for extensive periodontitis. She was effectively treated in collaboration with the hematology department.

**Case presentation:**

A 55-year-old Asian woman visited our department with concerns of worsening tooth mobility, discomfort, and spontaneous gingival bleeding. Initial periodontal examination revealed generalized severe periodontitis (Stage IV Grade C) resulting from leukopenia/neutropenia and poor oral hygiene. A thorough treatment strategy involving comprehensive dental procedures, such as multiple extractions and extensive prosthetic treatment, was implemented. Following the diagnosis of Felty syndrome, the patient was started on treatment with oral prednisolone 40 mg/day, which effectively controlled the disease. Furthermore, there was no recurrence of severe periodontitis after the periodontal treatment.

**Conclusions:**

Dentists and physicians should be aware that immunocompromised individuals with pancytopenia and poor oral hygiene are at risk of developing extensive periodontitis. If their susceptibility to infection and pancytopenia-related bleeding can be managed, such patients can still receive comprehensive dental treatment, including teeth extractions and periodontal therapy. Cooperation among the dentist, hematologist, and patient is necessary to improve treatment outcomes and the patient’s quality of life.

## Background

First identified in 1924, Felty syndrome (FS) is a severe variant of rheumatoid arthritis (RA) that is characterized by splenomegaly and neutropenia [1, 2]. FS usually develops in individuals with RA for more than 10 years and affects approximately < 1% of all RA patients [3]. In addition to autoimmunity, the presence of other risk factors has been suggested [3]; the exact cause, however, remains unknown. Some clinicians concentrate on severe extra-articular illness and neutropenia, which are associated with developing infections. However, the active extra-joint clinical characteristics in FS can be deceptive, and accurate diagnosis may occasionally be difficult. The complete triad of symptoms, namely neutropenia, RA, and splenomegaly, may not be present in an individual with FS. Neutropenia, defined as a neutrophil count < 1500/mm^3^, is a hallmark factor used to diagnose the disease [1]. We present a case of a patient with initially unapparent symptoms of marked leukopenia, splenomegaly, and RA, who developed severe periodontitis and FS.

## Case presentation

### Patient’s demographics

**Fig. 1:**
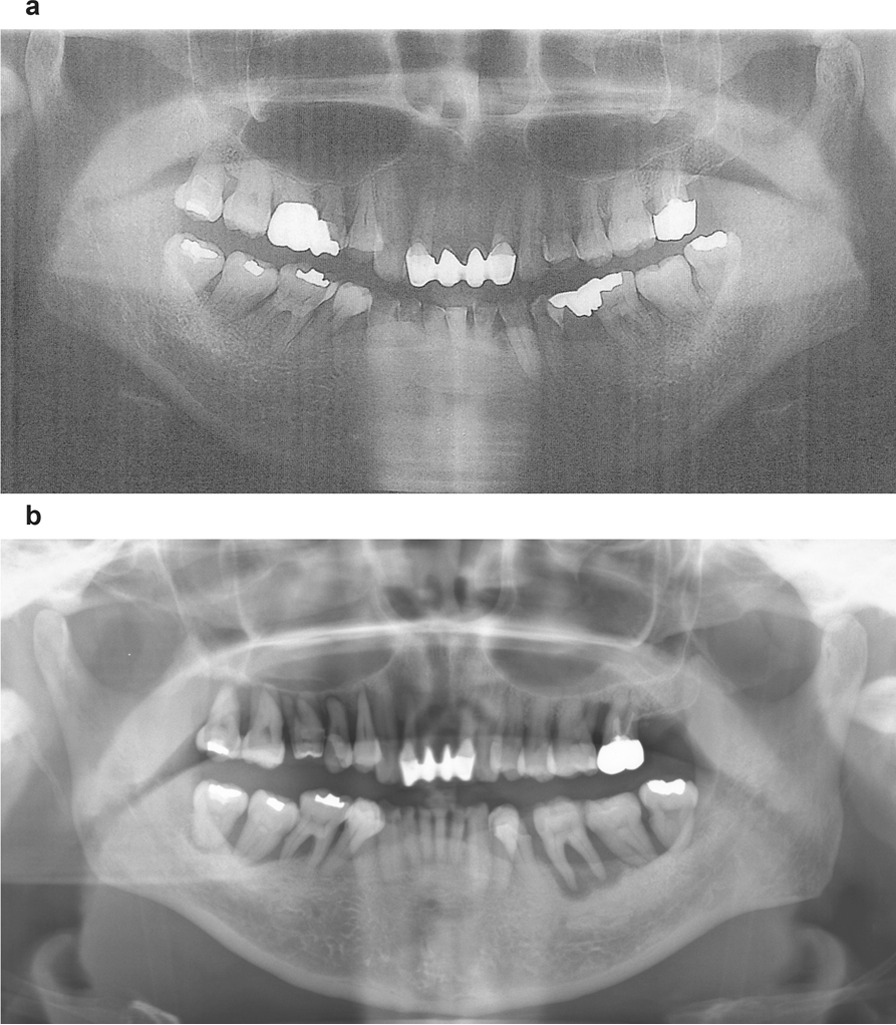
Panoramic oral radiographs reveal rapid alveolar bone loss after onset of Felty syndrome. **a** Panoramic radiograph taken 1 year and 5 months before the initial visit to our department. **b** Panoramic radiograph at the time of initial examination. Significant progression of alveolar bone resorption in a period of 1 year and 5 months is observed

A 55-year-old Asian woman with pneumonia was admitted to the hematology department of a general hospital in January 2016. Upon examination, leukopenia (0.5 × 10^3^/µL), anemia (hemoglobin, 7.0 g/dL), thrombocytopenia (133 × 10^3^/µL), and splenomegaly were observed. From the first visit, the patient showed symptoms of periodontitis that made oral intake difficult and thus resulted in anemia. Antimicrobial therapy was administered to the patient, and she also had pneumonia; however, a definitive diagnosis that could explain the pancytopenia and giant splenomegaly had not been established. The patient had been receiving regular dental care from her family dentist before she developed pancytopenia. The patient and her family confirmed the patient’s rapidly worsening periodontal disease over the year. We then obtained her previous panoramic radiographs, taken approximately 1 year and 5 months prior to referral. These radiographs revealed alveolar bone resorption in the maxillary and mandibular molar regions with the maximum resorption extending to half of the root’s length (Fig. 1a). A panoramic radiograph taken at our facility showed extensive and rapidly progressing alveolar bone resorption extending to the root apex and attachment loss in most maxillary and mandibular molars (Fig. 1b). The family dentist referred the patient to the Department of Oral and Maxillofacial Surgery at a university dental hospital, although the cause of the leukopenia was unknown at this point. The oral surgeon consulted at the university dental hospital found it difficult to provide dental treatment while managing the leukopenia and referred the patient to our department for tooth extraction and interdisciplinary management.

### Investigations

**Fig. 2:**
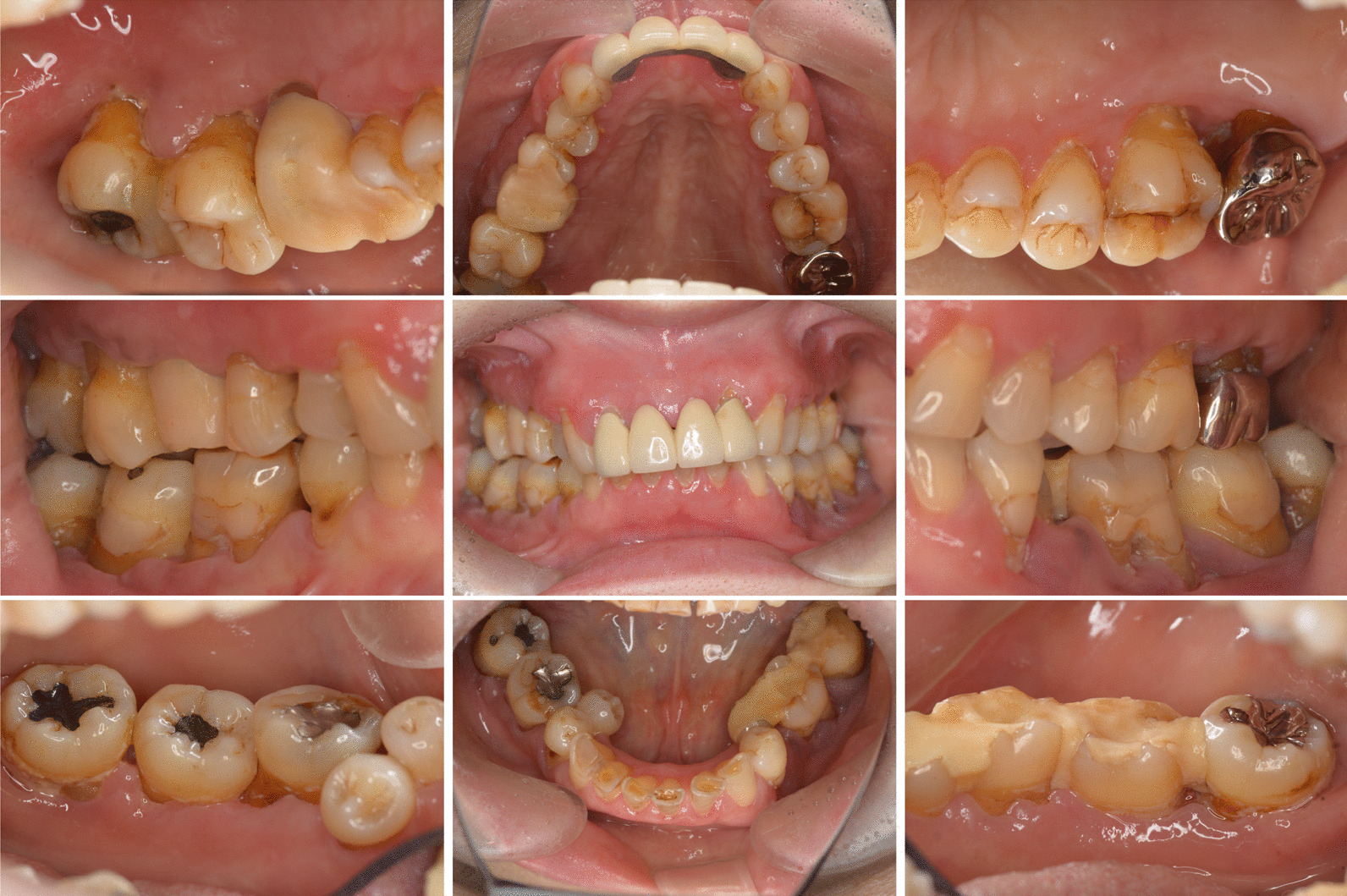
Clinical findings before therapy. Gingival edema and poor oral hygiene with poor restorations condition can be observed in the maxillary and mandibular regions, particularly in the bilateral molar regions

**Fig. 3:**
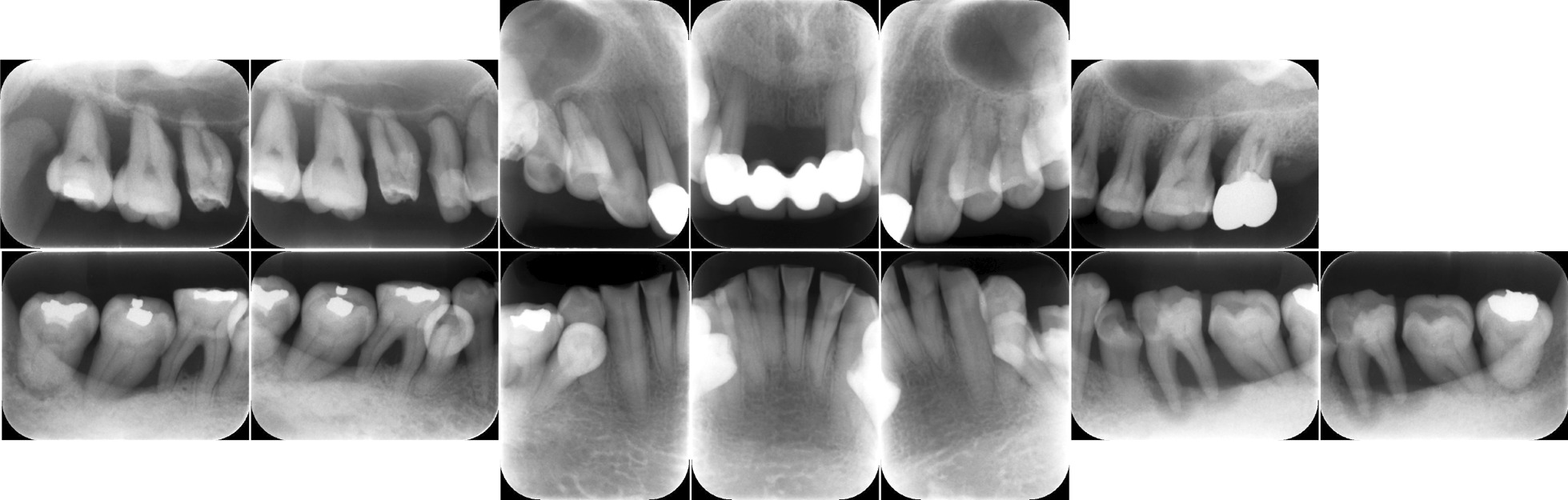
Initial periapical radiographs. Radiographs reveal widespread horizontal bone loss extending to the root apex

During the initial examination, the patient had poor oral hygiene and poor restorations on mandibular molars. The panoramic radiographs revealed horizontal and vertical bone resorption mainly in the bilateral maxillary and mandibular molar regions. Blood tests revealed leukopenia (0.6 × 10^3^/µL), 12% segmented neutrophils, anemia (red blood cell [RBC] count 256 × 10^4^/µL, hemoglobin 7.4 g/dL), and thrombocytopenia (103 × 10^3^/µL). After a thorough clinical examination of the periodontal disease and oral cavity, a treatment plan was developed based on the periodontal findings (Fig. 2). The periodontal pocket depth was measured at six points around each tooth to assess periodontal deterioration. Periapical radiography was performed to assess the extent of alveolar bone loss. Most molars showed severe alveolar bone loss extending into the apex area (Fig. 3). We confirmed deep periodontal pockets with probing pocket depths (PPDs) ranging from 4 to 12 mm. The periodontal inflamed surface area (PISA) was 3,210.6 mm^2^ [4]. A diagnosis of severe generalized periodontitis was made based on the findings of the initial periodontal examination (generalized Stage IV Grade C periodontitis) [5]. The onset of FS and poor oral hygiene, which may not be a major problem for dental patients with an otherwise normal immune status, caused severe and rapid alveolar bone resorption because of leukopenia and the consequent severe decrease in neutrophils, which are the chief immune cell population in healthy periodontal tissue [6–8].

We planned the extraction of more than 10 teeth and implementation of thorough oral hygiene protocols to prevent aggravation of the dental infections due to compromised immune function.

### Treatment

**Fig. 4:**
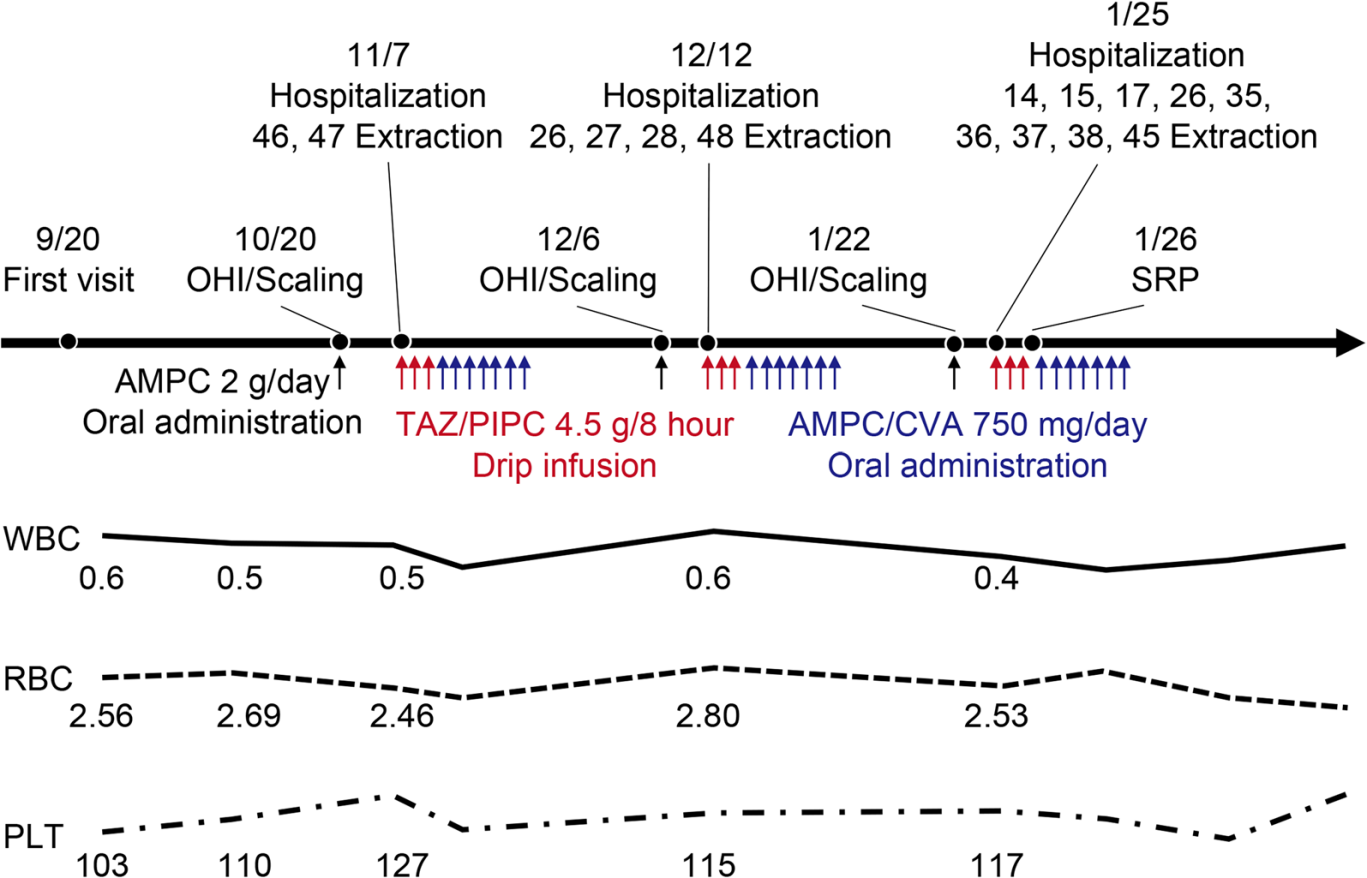
Flow of the treatment process during extraction of multiple teeth. Prior to tooth extraction, prophylactic antimicrobials were administered according to the guidelines. The patient’s first visit was on September 20, 2016. Administration of oral hygiene instruction and scaling were started on October 20, with 2 g oral administration of amoxicillin. Over 10 extractions were performed during the patient’s hospitalization, while administering intravenous antibacterial drip infusion of tazobactam/piperacillin (in doses of 0.5 g and 4.0 g, respectively) and amoxicillin/clavulanate (in doses of 250 mg and 125 mg, respectively) on November 7, December 12, and January 25. *OHI* oral hygiene instruction, *AMPC* amoxicillin, *TAZ/PIPC* tazobactam/piperacillin, *AMPC/CVA* amoxicillin/clavulanate, *SRP* scaling and root planning, *WBC* white blood cell, *RBC* red blood cell, *PLT* platelet

Since the cause of the pancytopenia had not been identified during planning, and postoperative infection was anticipated, intravenous tazobactam/piperacillin was administered to the patient. Over 10 extractions were performed during the patient’s hospitalization followed by basic periodontal treatment with detailed oral hygiene instructions, scaling, and root planing (Fig. 4). The cause of the pancytopenia was still undetermined at the end of the treatment. After extensive extraction of multiple teeth and basic periodontal treatment of the remaining teeth, the patient was discharged from the hospital, and treatment was continued on an outpatient basis. The anemia symptoms of the patient worsened during prosthodontic treatment with a crown, bridge, and removable partial denture; hence, the dental treatment was suspended, and she was admitted to the hematology department of a general hospital. An ultrasound examination of the hands and ankles revealed synovitis in the right knee and joint effusions in the ankle and knee joints; this was coupled with rheumatic signs, such as pancytopenia and joint pains. FS was diagnosed based on her previous leukopenia, splenomegaly, and the three main symptoms of chronic RA. Oral corticosteroid therapy (oral prednisolone 40 mg/day) was initiated immediately, and the pancytopenia improved. Additionally, blood test results indicated improved white blood count of 1.6 × 10^3^/µL, 64% segmented neutrophils, RBC count of 387 × 10^4^/µL, hemoglobin level of 13 g/dL, and a platelet count of 94 × 10^3^/µL.

### Outcome and follow-up

**Fig. 5:**
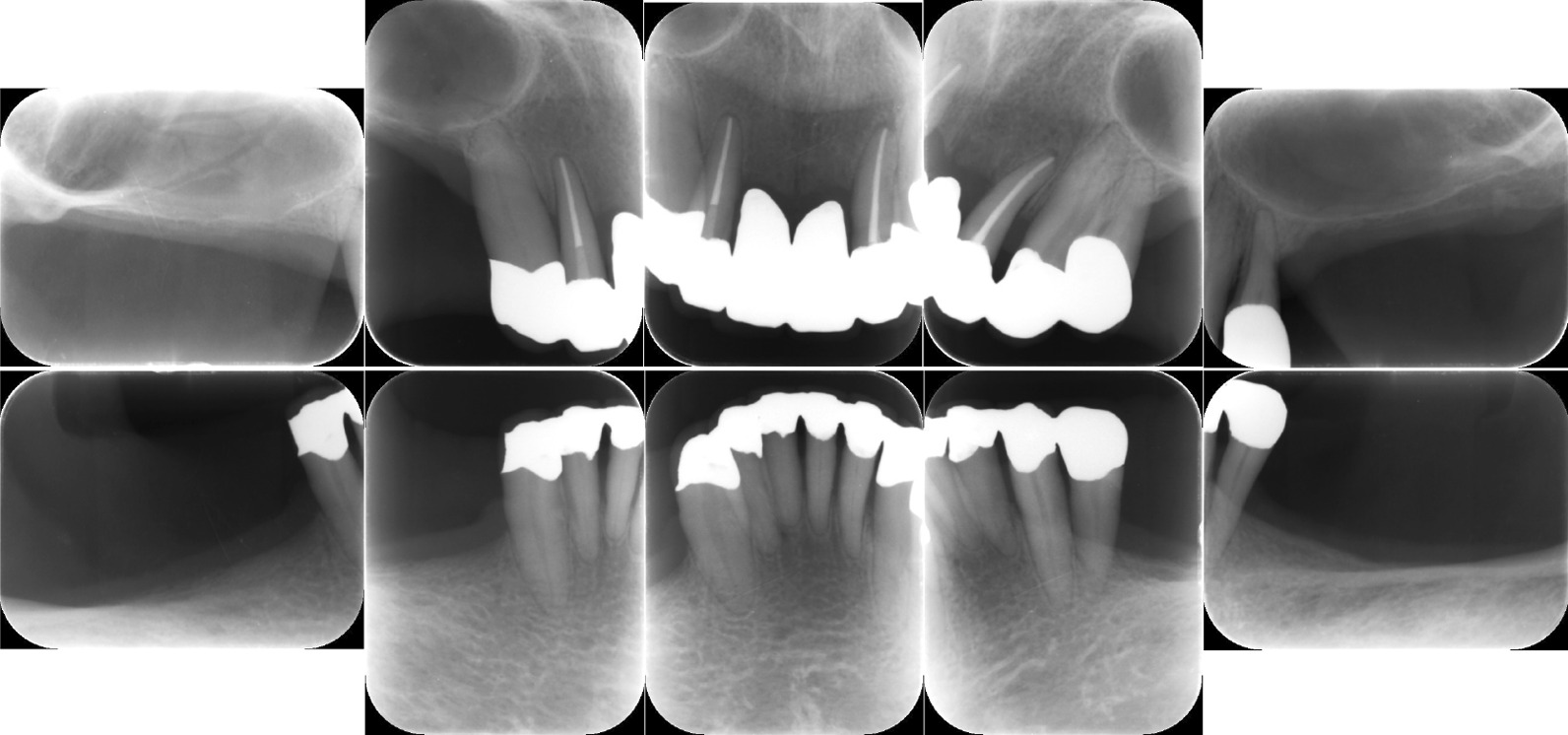
Periapical radiography reveals no sign of progression of bone resorption

**Fig. 6:**
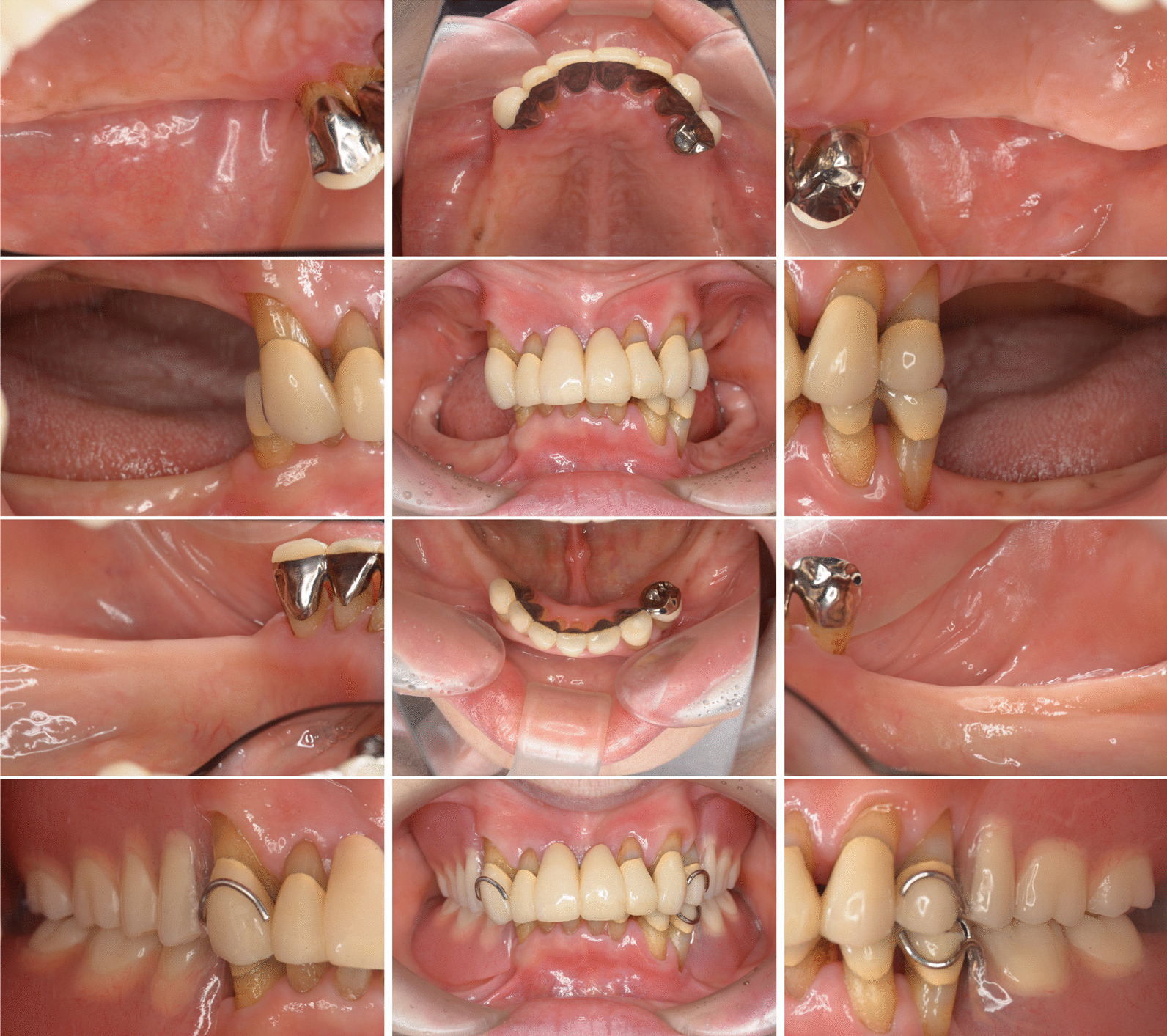
Intraoral pictures following complete treatment. No inflammatory findings are observed in relation to the remaining teeth

The patient returned for restoration of oral function with crowns, bridges, and partial dentures following improved pancytopenia. Following tooth extraction and basic periodontal treatment, the O’Leary plaque control record improved from 80.2 to 29.2% after treatment, the average PPD decreased from 5.7 mm to 2.5 mm, bleeding on probing reduced from 141 sites (83.4%) to 13 sites (18.1%), and the PISA decreased from 3210.6 mm^2^ to 100.8 mm^2^. Although all the molars were lost, there was no evidence of progressive alveolar bone resorption in the remaining teeth, as indicated by the dental radiographs (Fig. 5). Supportive periodontal therapy was initiated following treatment (Fig. 6). To date, there has been no recurrence of periodontitis with pancytopenia or bone resorption. The patient is currently receiving supportive periodontal therapy for periodontitis while being monitored for FS. There are no signs of relapsing periodontitis.

## Discussion and conclusions

The present case demonstrated the following. First, poor oral hygiene and poor restoration outcomes combined with FS can lead to severe generalized periodontitis characterized by extensive alveolar bone resorption within a short timeframe. Second, a synergistic medical and dental treatment approach is important, when FS is accompanied by neutropenia.

Periodontitis is generally triggered and maintained by a subgingival biofilm adhering to tooth surfaces. Dysbiosis and the ensuing polymicrobial disruption of the host’s homeostasis were believed to be the causes of late-onset periodontitis [9, 10]. In the present case, leukopenia in addition to poor oral hygiene and poor restoration outcomes on mandibular molars resulted in rapid progression of the periodontal disease. Early-onset periodontitis generally occurs in patients with neutropenia and aberrant neutrophils, which are crucial for the management of bacterial infection [8]. They are the effector immune cells in charge of the gingival antimicrobial defense and the initial line of defense against bacterial invasion. Neutrophils are essential for maintaining periodontal health as evidenced by the early-onset periodontitis that occurs in patients with neutropenia and aberrant leukocyte adhesions [11]. Most genetic disorders that predispose an individual to severe periodontitis have been linked to abnormalities in the neutrophil immune cell subset, indicating the major role of this immune cell subtype in maintaining periodontal homeostasis [12]. Oral neutrophils may provide the cell subsets involved in periodontitis because they are located at the interface between the periodontal tissues and the oral environment. In contrast with neutrophils present in healthy periodontium, sites affected by periodontal disease show many gene alterations in neutrophils [13].

According to recent research, polymorphonuclear leukocytes (neutrophils) are the primary immune cells driving periodontal disease progression [14, 15]. A previous study demonstrated that patients with periodontitis have an increased number of neutrophils in the oral cavity [16]. Furthermore, some investigations have demonstrated that, compared to healthy controls, peripheral blood neutrophils from individuals with periodontitis have an improved capacity to phagocytose and eliminate bacteria, generating noticeably more reactive oxygen species and neutrophil elastase [17–19]. These results show that patients with periodontitis have altered neutrophil function. Thus, neutrophils are important in periodontitis, and in FS, particularly with extremely low neutrophil count, characterized by tissue destruction due to rapidly progressing periodontitis, as in this case.

Pancytopenia is a transient condition that can occur in various hematologic and autoimmune diseases and as a symptom of myelosuppression caused by cancer chemotherapy. Therefore, it is likely to be encountered in clinical practice. In FS, pancytopenia is associated with bacteremia, making periodontal therapy challenging. A more comprehensive diagnosis and dental treatment plan, along with close collaboration with the medical staff are essential, for management of such cases. Further, the combination of poor oral hygiene, poor restoration outcomes, and onset of FS induces severe alveolar bone resorption in a short period and over a wide area, that is, generalized severe chronic periodontitis. Poor oral hygiene and poor restoration outcomes, which are not major issues in patients with normal immunity, combined with FS may exacerbate existing periodontal diseases. Since alveolar bone resorption is considered to have progressed rapidly after the onset of FS in this case, it is also considered diagnosable as “Periodontitis as a direct manifestation of systemic diseases” according to the new classification of periodontitis [20]. In our case, the cause of the perioperative pancytopenia, including neutropenia, was not determined, and strict postoperative infection control was considered necessary. First, the patient underwent preoperative oral prophylaxis indicated by oral hygiene instruction to improve oral hygiene. There have been no reports on the perioperative period in the field of dentistry in patients with FS. The patient responded well after receiving antimicrobial agents following the “Guidelines for the Appropriate Use of Antimicrobial Agents for Prevention of Postoperative Infection” and the “Guidelines for the Treatment of Febrile Neutropenia.” If pancytopenia is not correctly diagnosed and treated, periodontal pockets in cases of severe periodontitis can become infected with oral bacteria and cause severe systemic infections. Thus, chronic and acute infections must be treated and controlled appropriately in patients with pancytopenia, including FS.

Augustus Roi Felty defined FS as a triad of RA, neutropenia, and splenomegaly in 1924. An absolute neutrophil count and persistent neutropenia of typically < 1500/mm^3^ is required to establish a diagnosis even if the complete triad is not present [1]. Patients with RA who have unexplained splenomegaly and neutropenia are clinically diagnosed with FS. Sometimes, the signs of inactive joints cause clinicians to focus on neutropenia and extra-articular illness, which leads to recurring and sometimes fatal infections. Therefore, establishing the right diagnosis can be difficult, and FS is frequently mistaken for hematological malignancy. In the present case, FS was not diagnosed until after the surgical tooth extractions, and the white blood count ranged from 0.3 to 0.6 × 10^3^/µL. Comparison of the panoramic radiographs taken at the time of her visit to our department with the one taken by her family dentist 17 months earlier revealed rapid alveolar bone resorption. The patient was admitted to the hematology department in January 2016 with severe leukopenia and splenomegaly. Since rheumatic symptoms were absent, a definitive diagnosis of FS was not made.

In this case, all three criteria were present, and the characteristics of the systemic symptoms were consistent. Immunosuppressive therapy with corticosteroids is recommended for RA and leukopenia, and a strong antibacterial therapy is recommended for infection. The reason for the difficulty in the diagnosis of this case was believed to be the lack of RA symptoms and a focus on neutropenia alone.

In conclusion, severe periodontitis can be a significant outcome of pancytopenia combined with poor oral care. To prevent life-threatening situations and enhance the patient’s quality of life when both diseases co-exist, both the medical and dental teams need to work closely. We performed a pre- and postoperative antimicrobial administration procedure to prevent infection in this case of FS with neutropenia. Although this disease is relatively rare, it should be considered as a differential diagnosis when treating patients with leukopenia and neutropenia in clinical practice. Further, there are many causes of pancytopenia, and adequate collaborative measures with other relevant departments are necessary to prevent complications when performing invasive dental procedures. However, additional case studies and validation of this supposition are needed. Despite having severe chronic periodontitis and FS, a patient can still receive comprehensive dental therapy, including tooth preservation, extraction surgery, and comprehensive prosthetic treatment with control of the primary disease in collaboration with a hematologist, thus helping the patient achieve a better-quality lifestyle.

## Data Availability

All data generated or analyzed during this study are included in this published article. The dataset created during and/or analyzed during this case is available from the corresponding author of reasonable request.

## Abbreviations

FS: Felty syndrome
PPD: Probing pocket depths
RA: Rheumatoid arthritis
RBC: Red blood cell
PISA: Periodontal inflamed surface area
